# Age-Friendly Care Practices: Documenting Opportunities for Improvement

**DOI:** 10.1093/geroni/igaf122.497

**Published:** 2025-12-31

**Authors:** Taylor Bucy, Jessica Kalender-Rich

**Affiliations:** University of Kansas School of Medicine, Kansas City, Kansas, United States; University of Kansas School of Medicine, Kansas City, Kansas, United States

## Abstract

Efforts to address the multifaceted clinical and social needs of an aging population have centered around the Age Friendly Health System (AFHS) framework of 4Ms: Medication, Mentation, Mobility and what Matters. In 2025, hospital-wide AFHS structural measures were adopted by CMS, with both cost and quality implications. This study describes early efforts to document AFHS processes across a large Midwestern academic medical center. Using electronic health record data, we analyzed 447 unique patient encounters across two hospital units between July-September 2024. Mobility was measured using the bedside mobility assessment tool (BMAT), Mentation via the confusion assessment method (CAM) at least twice daily, Medication by use of antipsychotics, sedatives, and/or anticholinergics (i.e. monitored medications), and what Matters by person-reported location of expected discharge. On average, patients were 77.9 (+/- 6.3) years, with a median length of stay of 4 days (IQR=5) and an unadjusted 30-day readmission rate of 14.5%. The geriatric consult service saw 4.7% of patients. The majority (64.9%) of patients saw no improvement between admission and discharge BMAT. Roughly two-thirds of patients were administered a CAM screening twice daily, and 83.2% of patients received a monitored medication at least once. Finally, agreement between actual and expected discharge home was approximately 80%. Systematic tracking of age-friendly care facilitates early interventions aimed at standardization and prioritizes an environment of continuous learning. Our work identifies areas for targeted improvement prior to system-wide implementation of federal requirements and prompts further investigation into how geriatric consultation services can be leveraged to fill observed gaps.

